# A realistic model of pitch explains the N100m morphology evoked by dyads

**DOI:** 10.1186/1471-2202-16-S1-P213

**Published:** 2015-12-18

**Authors:** Alejandro Tabas, Emili Balaguer-Ballester, André Rupp

**Affiliations:** 1 Faculty of Science and Technology, Bournemouth University, Bournemouth, UK; 2 Bernstein Center for Computational Neuroscience, Heidelberg-Mannheim, Baden-Württemberg, Germany; 3 Heidelberg University, Baden-Württemberg, Germany

## 

Pitch is a fundamental attribute of auditory sensation underlying the perception of complex sounds. The N100m is a well-known transient neuromagnetic response of the evoked fields observed in magneto-encephalographic recordings, sensitive to fundamental properties of auditory stimuli such as pitch or timbre [1]. Despite considerable empirical evidence of the association between pitch perception and the N100m deflection in antero-lateral Heschl's gyrus (primary auditory cortex) [1], the neurophysiological mechanisms of pitch processing in cortex are still poorly understood. In this study we propose an innovative approach for understanding the pitch processing-N100m association by combining a biophysical model of the peripheral auditory system with a network of neural ensembles using realistic neural and synaptic parameters. The model is able to reproduce the morphology of the N100m component of auditory evoked fields in humans.

The model consists of three stages. First, a biophysically realistic model of the auditory periphery transforms the input stimuli into temporal patterns of auditory nerve activity [2]. This step is followed by an autocorrelation process that generates a spectro-temporal representation of the peripheral activity. These two stages are common in the literature and are supported by perceptual and neurophysiological data in sub-cortical areas [2]. The third stage processes patterns of brainstem activity by means of an ensemble model of 100 cortical populations parametrised by preferred frequency values [3]. The ensemble model stems from a mean field approximation of a network of spiking neurons, further simplified such that NMDA receptors dominate the model dynamics [3]; which yields to a network architecture with recurrent self-excitations and effective inhibitory currents between ensembles [3].

**Figure 1:**
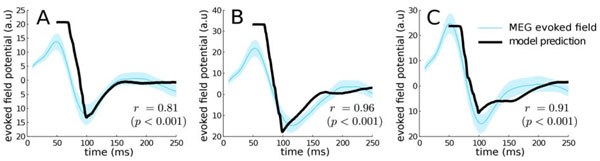
**Model predictions and recorded evoked fields for an unison dyad (A), a trinone (B) and a perfect fifth (C)**.

Electrophysiological predictions of the N100m morphology were derived from the aggregated dynamics of the gating variables of the cortical populations [4]; whilst the predicted pitch was encoded in a preferred frequency characterising a neural ensemble [2]. Biophysical parameters of the model were chosen according to neurophysiological data [4]. Mutual inhibition strengths, i.e. the effective connectivities between neural ensembles, were fitted and validated using pure and harmonic complex tones. The remaining parameters were tuned such that the model prediction for a unison dyad (two simultaneous harmonic complex tones) matched the evoked N100m morphology (10ms before and 70ms after the deflection). Model predictions were then evaluated using two other different dyads (see Figure 1).

The model successfully predicts the N100m morphology associated to such stimuli, for the first time to our knowledge. Furthermore, ensemble connectivities naturally reveal a harmonic structure critical for the cortical processing of pitch. Interestingly, the model dynamics unveils that that the N100m deflection is the result of the succession of a large increase in the input current of the neural populations followed by a selective inhibitory process. Thus, our results suggest that the model can potentially explain the biophysical mechanisms underlying a range of neurophysiological data associated to pitch perception.
